# Recent progress in paleontological methods for dating the Tree of Life

**DOI:** 10.3389/fgene.2012.00130

**Published:** 2012-07-13

**Authors:** Michel Laurin

**Affiliations:** UMR 7207, CNRS/MNHN/UPMC, Earth History Department, Muséum National d’Histoire Naturelle,Paris, France

**Keywords:** phylogenetics, molecular dating, molecular clock, paleontological dating, stratigraphic range, fossil record, confidence intervals

## Abstract

Dating the Tree of Life (TOL) has become a major goal of biological research. Beyond the intrinsic interest of reconstructing the history of taxonomic diversification, time-calibrated trees (timetrees for short, as used throughout below) are required in many types of comparative analyses, where branch lengths are used to assess the conservation importance of lineages, correlation between characters, or to assess phylogenetic niche conservatism, among other uses. Improvements in dating the TOL would thus benefit large segments of the biological community, ranging from conservation biology and ecology through functional biology and paleontology. Recently, progress has been made on several fronts: in compiling databases and supertrees incorporating paleontological data, in computing confidence intervals on the true stratigraphic range of taxa, and in using birth-and-death processes to assess the probability distribution of the time of origin of specified taxa. Combined paleontological and molecular dating has also progressed through the insertion of extinct taxa into data matrices, which allows incorporation of their phylogenetic uncertainty into the dating analysis.

## INTRODUCTION

### WHY DATE THE TREE OF LIFE?

Some of the most basic questions about the evolution of life concern the chronology of events. When did a given taxon appear? When did it diversify? Was its diversification slow and gradual, or did it occur in bursts (evolutionary radiations), and if so, when were these bursts, and what caused them? Answering such questions is important not only to satisfy our intellectual curiosity about the history of life, but also to allow sophisticated analyses in other fields.

Dating the Tree of Life (TOL) has become a major goal of biological research, as shown by various well-funded initiatives (at least in the USA), such as the Assembling the Tree of Life program of the National Science Foundation http://www.nsf.gov/funding/pgm_summ.jsp?pims_id=5129, which has distributed over US $ 57 000 000 since 2006. Dating the TOL has also been the exclusive subject of a large, recent edited book (Hedges and Kumar, 2009a), and some molecular systematics laboratories now specialize in this task. This interest in dating the TOL is not surprising because beyond the intrinsic goal of reconstructing the history of taxonomic diversification, timetrees are required in many types of comparative analyses. In fact, time data are so useful that Avise and Liu (2011) have suggested to systematically add this information to taxon names.

These advances in evolutionary biology, which require timetrees, have revolutionized modern science by allowing more rigorous analyses. For instance, conservation studies previously used species counts (at best) or higher taxon count (of genera, families, or even orders) to assess the biodiversity of various regions (hotspots or simply areas that could be protected to preserve as much biodiversity as possible), but all these approaches are problematic to various extents. There is no universally accepted species concept, despite repeated attempts to unify species concepts (e.g., de Queiroz, 1998). As such, the entities that we call “species’’ and that are registered into various databases are not ontologically comparable; some are clades, some are reproductive communities, some are evolutionary lineages, others are phenetic clusters, and yet others belong to two or more of these categories. This problem has far-reaching consequences for conservation biology (Bertrand et al., 2006), and it has hampered developments of a new, phylogenetic code of biological nomenclature for species (Laurin and Cantino, 2007). Taxa belonging to higher categories are even more problematic in conservation biology because, like species, they share no objective properties, and they often differ rather strongly in many important aspects, such as geological age of origin, number of included species, or phenotypic variability (Laurin, 2005, 2008; Bertrand et al., 2006). The most objective solution that has been advocated (and widely applied) to circumvent all these problems is to use phylogenetic diversity (the sum of branch lengths expressed as time), as this captures the sum of the unique history of a given clade, or of a given fauna or flora of a region (Faith, 1992).

In comparative biology, timetrees have become essential since we have realized that comparative data are not statistically independent from each other. This is because closely related taxa tend to resemble each other more closely than distantly related taxa. Thus, all modern comparative techniques for inter-specific datasets incorporate phylogenetic information, ideally in the form of timetrees. The interest of this approach is demonstrated by the high number of ISI citations (1371) of the paper by Felsenstein (1985a) that presented the method of phylogenetically independent contrasts (ISI databanks consulted on March 4, 2011). Similarly, the related method called phylogenetic generalized least squares (PGLS; Martins and Hansen, 1997; Pagel, 1997) is increasingly used in such studies (e.g., Spoor et al., 2007; Organ et al., 2011a,b). Branch lengths are also needed to study phylogenetic niche conservatism (Patterson and Givnish, 2002; Desdevises et al., 2003; Losos, 2008), evolutionary trends from paleontological data (Laurin, 2010a), or to determine ancestral conditions through methods such as squared-change parsimony (e.g., Laurin, 2004; Finarelli and Flynn, 2006), Maximum Likelihood (Pagel, 1999), or Bayesian inference (e.g., Organ et al., 2011a,b).

Various evolutionary problems, such as the search of a correlation between past climatic changes or geological events (i.e., the break-up of Pangea) and taxonomic or phenotypic diversification, require timetrees to be meaningfully studied (e.g., Paradis, 2004; San Mauro et al., 2005). Thus, timetrees are at the core of several biological fields, so any project that would significantly improve how they are built would benefit a large segment of the biological community.

From an economic perspective, the applications of timetrees in conservation biology (Faith, 1992), through better-informed choices in conservation priorities (of regions or taxa), are probably the most important. Indeed, the twenty-first century faces a major biodiversity crisis and may well be the “century of extinction’’ (Dubois, 2003), in addition to (and partly because of) facing climate change caused by greenhouse gas emissions. Biodiversity is important for the maintenance of ecosystems, and the crucial role of “ecosystem services’’ (in maintaining a climate favorable to agriculture and human health, in absorbing greenhouse gases, etc.) are now beginning to be better documented (Heikkinen et al., 2006; Eronen et al., 2010), even though policy-makers have not yet understood the importance of preserving old species-poor clades such as the coelacanths, lungfishes, and monotremes (Lecointre, 2011). Therefore, it is very important to develop better methods for calibrating molecular timetrees.

### HISTORICAL DEVELOPMENT OF TIMETREES

Historically, dating the TOL relied initially almost exclusively on the fossil record (e.g., Hennig, 1981). Paleontologists inferred the relationships between various taxa using their morphological similarities, and the fossil record provided directly minimal divergence times between taxa. Actual divergence times were of course acknowledged to pre-date the oldest fossils attesting to the presence of a taxon, with the delay between actual appearance and first fossil record being vaguely proportional to the morphological gap between the taxon of interest and its presumed predecessors (Romer, 1966). Thus, it was widely admitted that the evolution of taxa lacking a fossil record was more or less impossible to reconstruct (e.g., Gingerich, 1979, p. 454). In the last decades, the introduction of cladistics (Hennig, 1966) in paleontology has resulted in rapid progress in our understanding of the phylogeny of various taxa, sometimes resulting in rather radical changes in our ideas (e.g., Janvier, 1996), or in new heated debates about the origin of various extant taxa, such as turtles (Reisz and Laurin, 1991; Lee, 1995; deBraga and Rieppel, 1997; Lyson et al., 2010) and lissamphibians (Ruta and Coates, 2007; Anderson et al., 2008; Marjanović and Laurin, 2009). 

More recently, use of the fossil record has increasingly been complemented by molecular timetrees, a change allowed by the tremendous growth of molecular phylogenetics in the last two decades (e.g., Sanderson, 2003; Hedges and Kumar, 2009a), even though the roots of molecular dating hark back to the 1960s (Zuckerkandl and Pauling, 1962). The basic principle of molecular dating rests on inferring how much molecular change has occurred on each branch of the reconstructed tree (or trees, when Bayesian methods are used). This is not straightforward because multiple changes can occur at a given nucleotide site, and given that there are only four possible states (A, T, C, or G), a given site may revert to its initial condition. Furthermore, all sites do not evolve at the same speed; some genes evolve faster than others, mitochondrial genes tend to evolve faster than most nuclear genes, silent portions of the genome tend to evolve faster than functionally important portions, and third codon positions evolve faster than first and second codon positions because many changes in the third position result in the same amino acid. Thus, molecular biologists have developed various evolutionary models that attempt to account for these factors, as well as sophisticated methods to select the best-supported model (e.g., Posada, 2008).

The resulting tree is usually not ultrametric because each lineage evolves at its own rate; thus, the tips (extant terminal taxa) are each at a slightly different patristic distance from the root of the tree. The differences in height represent deviations from the hypothesis of a molecular clock. The tree can be ultrametricized using various algorithms, which, in a best-case scenario, results in a tree in which branch lengths are proportional to time. However, to be converted into absolute time, such trees need to be calibrated by dating some cladogeneses (divergences) through the fossil record (if fossils indicate the approximate time at which a given divergence occurred) or geological events (like separation between continental plates, under the hypothesis of vicariance). Multiple calibrations are best because these can document the changes in molecular evolutionary rates and the improved rate evolution modeling should improve the dating of the tree (Britton, 2005). However, as we will see below, getting these calibration data is usually the limiting factor in accurately dating the TOL. This paper reviews recent progress in methods to better get these constraint data and to make them more readily accessible.

### CURRENT LIMITATIONS FOR TIMETREE CONSTRUCTION

#### Methodological limitations

Because of the recent rise in molecular systematics, paleontological data have become more and more neglected. For instance, the largest share of grant support given in the context of the “Assembling the Tree of Life’’ program of the National Science Foundation went to molecular systematics in the broad sense (possibly incorporating some DNA barcoding); little was given to paleontology. The large edited book on the TOL (Hedges and Kumar, 2009a) was written mostly by molecular systematists (a few paleontologists contributed to a few chapters) and was based largely on molecular dating. We have now reached a point at which progress is limited by the availability of paleontological data to calibrate the molecular timetrees (Brochu, 2004; Britton, 2005; Marjanović and Laurin, 2007; Sauquet et al., 2012).

Other steps of the molecular dating process have been much more intensively financed and have attracted much more attention. These steps include gathering data on extant biodiversity (a major preoccupation of systematists since the eighteenth century), sequencing various genes of these taxa (a blossoming, well-financed field, at least in some countries), analyzing these sequences (to align them, to select appropriate nucleotide substitution models, etc.), and using molecular dating methods. Several systematists have worked on the latter (e.g., Sanderson, 2002; Thorne and Kishino, 2002; Li et al., 2011) and have now produced methods so sophisticated (e.g., Drummond and Rambaut, 2007) that they can use data that we are only beginning to get for a few clades (Wilkinson and Tavaré, 2009; Wilkinson et al., 2011), namely not only most probable (rather than minimal) divergence date constraints, but also variances around these point estimates, and even the shape (normal, log-normal, exponential, asymptotic, etc.) of the prior distribution around these point estimates (Ho and Phillips, 2009). Strangely, the fact that we lack these critically important paleontological data has apparently not preoccupied most systematists; very few efforts have been made to solve this problem (; Marjanović and Laurin, 2007, 2008), and we are still far from an adequate, generally applicable solution (Parham et al., 2012). Thus, working on this problem should constitute by far the most cost-effective solution in improving dating of the TOL. 

This situation is unfortunate because molecular dates depend heavily on dating constraints, and the fossil record provides the best constraint data (Brochu, 2004; Marjanović and Laurin, 2007; Lee et al., 2009; Inoue et al., 2010). Geological events, such as the separation between various continental plates, can also be used, but with a greater risk of error because the assumption that the distribution of extant taxa reflects vicariance is not always justified, because the dates of separation of continental plates are often poorly constrained, because land bridges, island chains, and channels can act as dispersal routes, and because the extant distribution of a taxon may not reflect earlier distributions (Ho and Phillips, 2009). For instance, Phillips et al. (2010) concluded that the apparent Gondwanan distribution of ratites is artifactual and that multiple flight losses in the group imply that they may have dispersed far more easily than previously thought. Similarly, Pascual et al. (1992) showed that monotremes, which are restricted today to Australasia, once occurred in South America. Thus, biogeogeographic data should be used only very cautiously to constrain molecular dating studies, and unfortunately, the taxa for which they might be the most useful (those with a scanty fossil record) are precisely those for which using biogeographic data is most dangerous because there is no way to determine if the present geographical distribution of such taxa reflects their past distribution. Because of this, paleontological data constitute the best direct source of calibration to date the TOL.

Brochu (2004) showed that molecular ages inferred from quartet dating display a strong dependence on the age of the calibrations used, even if the age of each calibration is correct. Using crocodylians as a test case (which is appropriate given their dense fossil record and their intensively studied phylogeny), Brochu (2004) showed that using two recent (e.g., Neogene) events to infer the age of the divergence between crocodylids and alligatorids consistently under-estimated the age of this event, which the fossil record indicates occurred about 78 Ma ago. Conversely, using old calibration constraints (e.g., Paleogene) consistently over-estimated the age of this divergence (to over 100 Ma ago, sometimes over 200 Ma ago). This phenomenon is unexpected because under the original formulation of the principles of molecular dating (Zuckerkandl and Pauling, 1962), any calibration constraint could be used, and provided that the constraint is correct, results should be independent of the constraint used. This problem could perhaps be dismissed if it could be shown to be limited to one dataset or to quartet dating, which has been replaced by more sophisticated methods, such as penalized likelihood (Sanderson, 2002, 2003) and Bayesian inference (e.g., Drummond and Rambaut, 2007). An explanation for this phenomenon was proposed by Hugall et al. (2007), who showed that saturation of DNA sequences and the consequent compression of basal branches leads to overestimation of node ages if only external deep calibrations are used. Thus, this problem is not restricted to quartet dating, and the most effective solution is likely to be using more calibration constraints distributed in various parts of the tree. 

Even the shape of the prior distribution around the dating constraints influences strongly molecular dates. Ho and Phillips (2009) demonstrated this using an amniote dataset emphasizing neornithine birds. Using three constraints (divergences between lepidosauromorphs and archosauromorphs, between paleognaths and neognaths, and between penguins and loons), they demonstrated that using point-like estimates spanning only 1 Ma toward the youngest age compatible with the fossil record, the basal neognath node could be dated from the early Cenozoic (approximately between 60 and 65 Ma). Using widely spread maximal and minimal hard bounds for these three time constraints, the posterior distribution of ages shifted entirely into the Mesozoic (roughly, from 66 to 113 Ma). Using a lognormal prior distribution adjusted so that 95% of the prior distribution fell within the hard bounds, the posterior distribution was slightly shifted toward more recent times than using hard bounds, but over 95% of the posterior distribution remained within the Mesozoic. The spectacular difference between the ages yielded by the three ways of specifying the time constraints illustrates the importance of this step in the analyses. The only problem in the demonstration of Ho and Phillips (2009) is that few (if any) paleontologists would advocate setting any maximal age constraint within 1 Ma of the age of the oldest fossil pertaining to a clade, even with a fairly dense fossil record. Previously, for lissamphibian taxa with the densest fossil record, Marjanović and Laurin (2007) did a series of analyses with maximal ages set 15, 30, and 45 Ma from the minimal age.

Hug and Roger (2007) and Marjanović and Laurin (2007), among other studies, demonstrated the extreme importance of maximal age constraints in molecular dating. Marjanović and Laurin (2007) demonstrated that the molecular ages yielded by penalized likelihood on a dataset composed of whole mitochondrial genomes of lissamphibians depend most heavily on the use of maximal bounds on calibration constraints. This result probably applies to Bayesian dating as well because by using the same set of calibration constraints, Marjanović and Laurin (2007) reproduced the results initially obtained by Zhang et al. (2005) using MultiDivtime (Thorne and Kishino, 2002). Thus, while the analytical method or evolutionary model selected had only a modest influence on the obtained ages, Lissamphibia could be as old as Early Carboniferous (321–356 Ma ago, in this case) if no maximal age constraints were enforced, or as recent as Permian (250–291 Ma ago, in this case), if a few maximal age constraints were enforced. This explains that some molecular ages for Lissamphibia are even older, and may explain the striking differences between molecular and paleontological ages of several taxa (Yamanoue et al., 2006; Tinn and Oakley, 2008; Inoue et al., 2010). For instance, Roelants et al. (2007) inferred a Devonian age (368.8 Ma ago) for Lissamphibia, which implies a gap of 120 Ma in the lissamphibian record, as the oldest known lissamphibians are from the Early Triassic, less than 250 Ma ago (Rage and Rovc, 1989; Evans and Borsuk-Bial, 2010). However, this surprising result appears to be attributable to the use of numerous minimal internal age constraints (15 paleontological and seven geological), while no maximal internal age constraint was enforced (Roelants et al., 2007: SOM Figure 4); only the ages of Tetrapoda and Amniota had minimal as well as maximal age constraints. This was no accidental choice because several molecular biologists (e.g., Hedges and Kumar, 2009b) have argued that maximal age constraints can rarely be know. However, given the findings reported above, using several minimal time constraint and only one or a few maximal age constraints is a recipe for overestimation, as if curve-fitting were performed by minimizing the square of the distance between the curve and the data points only in one direction. The development of recent software that allows soft bounds (in which prior probability that a divergence occurred before the soft bound is small and decreases progressively) to be used (e.g., Drummond and Rambaut, 2007; Ronquist et al., 2012) should minimize this problem, provided that the prior distribution is not too broad (which would result in large credibility intervals).

Hug and Roger (2007) demonstrated similar effects on metazoan dating using non-parametric rate smoothing (NPRS), penalized likelihood (PL), and Bayesian dating with linked and unlinked branch lengths. Unreasonably old ages were yielded by the use of very old maximal ages, with the age of Metazoa being estimated at up to about 2.3 Ga with NPRS, when the (less inclusive) constraints were only specified to be less than 1.5 Ga. They assessed the relative importance of several factors in determining the molecular dates and concluded that the influence of the calibration ages is the greatest, and that maximal age constraints are required, even though they are much more difficult to get than maximal age constraints. They also concluded (Hug and Roger, 2007, p. 1893) that using a single calibration constraint results in the “significant introduction of error into the age estimates.’’ Other factors, such as taxonomic sampling of sequenced lineages or analytical method (NPRS, PL, Bayesian) had much less influence on molecular dates. They concluded that the best dating strategy was to maximize the number of reliable and reasonably narrow calibration constraints, rather than to maximize the number of gene sequences included. Thus, obtaining maximal age constraints is of paramount importance to obtain accurate molecular dates.

**FIGURE 1:**
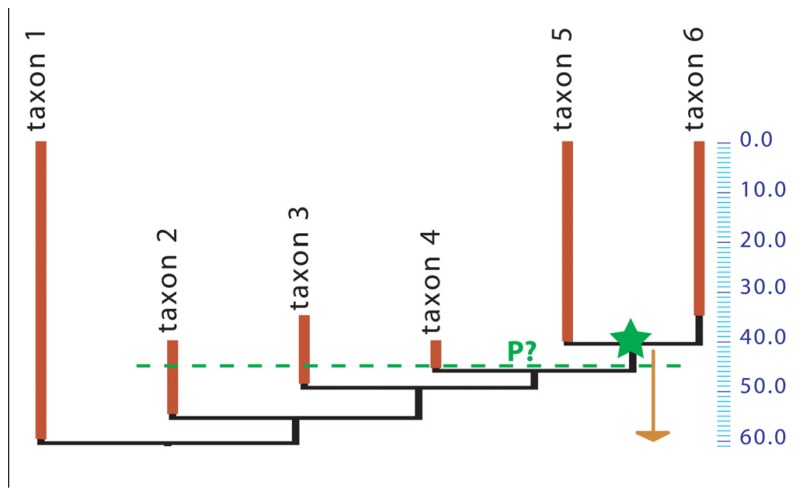
**Phylogeny of a hypothetical taxon.** A crown group (containing taxa 5 and 6) appears in the fossil record 40 Ma ago (green star) but the inferred molecular age is 60 Ma (arrow). The green dashed line at 45 Ma ago shows that three stem-taxa are observed at that time. All taxa shown here may represent a single evolutionary lineage (species, under some species concepts) or clades that may include several species. What is the probability (P?) that the crown group existed 45 Ma ago?

Unfortunately, while obtaining minimal divergence dates from the fossil record requires only placing the relevant fossils in a phylogeny, getting maximal age constraints remains an outstanding problem, arguably the greatest remaining methodological challenge in dating the TOL. Getting accurate time constraints is now the limiting factor because no generally applicable, rigorous method has been developed to estimate the most probable and maximal divergence dates of taxa based on the fossil record (Ksepka et al., 2011), even though several studies have developed methods to assess the reliability of dating constraints (e.g., Near et al., 2005; Pyron, 2010). Several estimates of maximal ages rely on the age of fossils located on the stem of the crown-group to date (e.g., Reisz and Müller 2004; Müller 2005; Marjanović and Laurin and Laurin, 2007, and references cited therein). However, this approach is neither rigorous nor widely applicable, because of the notorious incompleteness of the fossil record. For instance, suppose that a given crown clade appears in the fossil record 40 Ma ago, with three successive sister-taxa on the stem of that clade appearing at 46, 49, and 55 Ma ago (respectively), but that the inferred molecular age of the crown clade is 60 Ma (**Figure 1**). Is this pattern of distribution of fossils coherent with the inferred molecular age? What is the probability of such a stratigraphic distribution of fossil finds if the molecular age is true? Nobody knows for sure. No method can give the probability that the crown-clade existed at 55 or 60 Ma ago (for instance) from such paleontological data alone, although the method of Wilkinson and Tavaré (2009) can be used when abundant data on diversity through time are available. If we used a simple binomial distribution and assumed that every lineage had an equal chance of preservation, our estimate of the probability that the crown-clade existed (without being observed) 45 Ma ago, given that multiple stem-lineages are observed at that time, would depend on the estimated paleobiodiversity of the stem-members of the clade. Unfortunately, there is no simple way to assess the unobserved paleobiodiversity. 

#### Limitations linked with the fossil record

The problem of estimating the maximal age of clades based on the fossil record has been frequently discussed because if it of paramount importance in molecular dating. Ho and Phillips (2009, p. 374) listed five sources of uncertainty that must be taken into consideration when tackling this problem: (1) fossil preservation; (2) taxonomic assignment of fossils; (3) identification of fossil homologies; (4) sampling effort, and (5) fossil age determination. These factors can be sorted into three groups because fossil preservation and sampling effort are related because what really determines our knowledge of the fossil history of a taxon is the fossil collections, whereas taxonomic assignment is determined largely by the fossil homologies, so these are two sides of the same coin. 

Fossil preservation and sampling effort are the most difficult problems. For some large clades, these factors can be effectively solved by using an exponential model of diversification (e.g., Marjanović and Laurin, 2008), but in many cases, the problem is likely to be much more complicated by several factors that may bias the fossil record. Thus, taxa that lack mineralized parts (many annelids, nematodes, etc.) often have a very poor fossil record (Laurin, 2010b), although there are obvious exceptions, such as embryophytes, which have an abundant fossil record. The global fossil preservation rate is difficult to quantify, as shown by the fact that it has been estimated to be as low as 1–5% by Forey et al.(2004, p. 642), and possibly as high as 38% (both at the species level) by Foote, 1996: Table 1). However, it is clearly much higher, possibly up to 90% complete for taxa that have mineralized structures, at least for periods in which fossiliferous rock sequences are preserved (Foote, 1996: Table 2).

Ecological biases affect the fossil record because most fossils are derived from remains that were deposited in aquatic environments. Thus, taxa inhabiting arid environments are under-represented in the fossil record. Similarly, alpine faunae and florae are poorly known because sediments deposited in high-altitude lakes and rivers are likely to be quickly eroded. Indeed, several Paleozoic fossiliferous localities that have long been interpreted as freshwater “intra-montane basins’’ show signs of marine influence, so if there were mountains nearby, they must have been coastal, and the preserved environments were obviously near the sea level (Laurin and Soler-Gijón, 2010). A few famous examples include Joggins, Nova Scotia, from which the oldest amniote remains were recovered. Long interpreted as intra-montane, it has been shown to harbor marine or brackish-water taxa, such as xiphosuran chelicerates (Falcon-Lang et al., 2006). Another spectacular example is Montceau-les-Mines and surrounding Massif Central permo-carboniferous localities, which were long interpreted as intra-montane basins, with geologists even arguing for very high altitudes of 4500–5000 m above sea level (Becq-Giraudon et al., 1996, p. 239). Yet, recent paleontological investigations have shown the presence of marine taxa in Montceau, such as hagfishes (Poplin et al., 2001). Even marine environments are affected by their own biases because epicontinental seas have varied in extension following sea level fluctuations, whereas continental drift has “recycled’’ most pre-Jurassic oceanic plates, leading to a virtual absence of pre-Jurassic fossil record of deep-sea environments. Thus, fossil preservation is strongly habitat-dependent.

Geographic biases also affect the fossil record. Thus, fossiliferous strata in rich, stable countries where several paleontologists work are probably much better-studied than those of less prosperous and less politically stable countries. In fact, there are many more fossiliferous outcrops than can be prospected by paleontologists, and there is no reason to think that what gets prospected is a representative (random) sample of the available exposures. Modern environments also create biases because some environments, such as warm, humid environments like the Amazonian jungle are very unfavorable to the discovery of fossils because a thick layer of organic-rich soil covers the rock exposures, whereas others, like deserts, are notoriously fossiliferous because the absence of vegetation cover facilitates detection of fossils. The combination of ecological and geographic biases mean that even within mid-sized clades, the fossil record is of heterogeneous quality. Empirical studies support this theoretical expectation. Thus, Marjanović and Laurin (2007: Figure 9b) concluded that the age of some lissamphibian taxa, like Urodela, could be reasonably well-constrained by the fossil record (to about 155–170 Ma), whereas others, like Neobatrachia, could not (range of plausible ages from about 70–130 Ma). This is not surprising given that basal urodeles include many aquatic to amphibious taxa that are expected to have a good fossilization potential, whereas neobatrachians include several terrestrial forms, some of which live in high altitude or have recently diversified in tropical environments, where the potential to recover fossils is low. In fact, the Mesozoic fossil record of lissamphibians shows a rather strong bias toward aquatic taxa, such as cryptobranchid and sirenid urodeles, or pipid anurans. The recently described oldest (stem-) salamandroid, *Beiyanerpeton jianpingensis* (Gao and Shubin, 2012) is no exception as this taxon shows traces of a lateral-line organ, had a deep tail, and various paedomorphic characters, all of which indicate an aquatic lifestyle. 

Taxonomic uncertainty is generally greater with extinct than extant taxa, although since the advent of cladistics (Hennig, 1966), much progress has been done on this front. Hennig introduced several new ideas into phylogenetic research, one of which is that it is almost impossible to positively identify actual ancestors because such an identification rests on negative evidence. This is because the presence of autapomorphies (unique derived character states) demonstrates that a given taxon is not the ancestor of any other taxon that lacks these apomorphies, but the absence of known autapomorphies in a fossil could simply result from non-preservation of the relevant characters. Thus, many paleontologists consider that we have probable ancestor-descendant sequences only in cases of densely sampled diachronous populations from a given locality, which usually document mostly infraspecific evolutionary patterns. Consequently, most paleontologists have given up the search for ancestors and concentrate on assessing sister-group relationships (e.g., Müller 2006; Ruta and Coates, 2007; Laurin, 2010b). This complicates using the fossil record to assess divergence times because the presence of an ancestor of a taxon at a given time would prove that the taxon had not yet diversified, which would validate use of that fossil to set maximal age limits for molecular dating. However, the presence of one or even several sister-groups of a taxon at a given time does not prove that the taxon had not started diversifying. This explains why it is so difficult to use the fossil record to set maximal age constraints (Müller 2005; Marjanović and Laurin, 2007). Nevertheless, the introduction of cladistics and other modern phylogenetic methods in paleontology now allow uncertainty to be quantified through bootstrap values (Felsenstein, 1985b; Sterli et al., 2012) or posterior probabilities (e.g., Müller 2006). The recent development of non-invasive 3D imaging techniques now allows extracting much more information from some fossils, which will also contribute to producing more detailed and reliable phylogenies in the future (e.g., Pradel et al., 2010); these techniques have recently produced sufficiently new data to result in the publication of special issues of scientific journals (e.g., Clément and Geffard-Kuriyama, 2010). 

Fossil age uncertainty is highly variable and is mostly locality-dependent. Some localities are dated fairly precisely through radiometric dating, whereas others are less well-constrained and are only loosely correlated with other radiometrically dated localities through biostratigraphy (Gradstein et al., 2004a). However, geologists are making steady progress toward dating the sedimentary rocks, and are even refining the geological timescale and how it is defined, for instance through the use of GSSPs (Global Stratotype Section and Point) that replace the classical type-sections by precisely defining the lower boundary of each time unit (Gradstein et al., 2004b).

To sum up, rigorously computing the probable and maximal appearance dates from a paleontological tree, or from paleontological data more generally, is a complex (but probably solvable) mathematical problem that will require substantial methodological developments and data acquisition to effectively tackle. Fortunately, progress is being made in this direction, and these developments are reviewed below. This review frequently uses the example of lissamphibians to illustrate many of the relevant concepts and problems, but this simply reflects my interests and competence. Thorough dating studies have been published for many other taxa, such as embryophytes (e.g., Magallón, 2010), eukaryotes (e.g., Hug and Roger, 2007), metazoans (e.g., Peterson et al., 2004), birds (e.g., Dyke and van Tuinen, 2004; Brown et al., 2008), or placental mammals (e.g., Bininda-Emonds et al., 2007; Meredith et al., 2011), among other examples.

## RECENT METHODOLOGICAL PROGRESS IN DATING THE TOL

### DATABASES, COMPILATIONS, AND SUPERTREES

**FIGURE 2:**
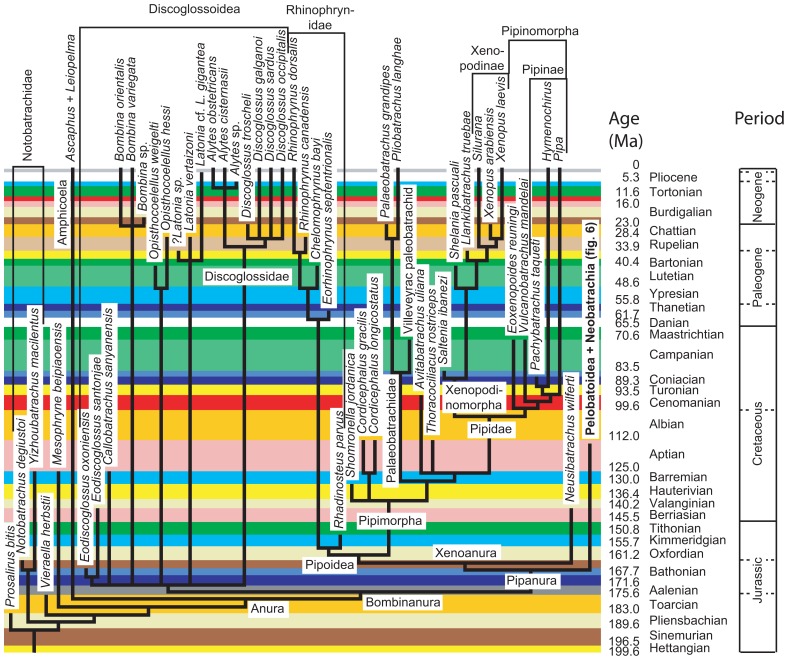
**Part of the time-calibrated supertree of lissamphibians presented by** Marjanović and Laurin (2007. Modified from Marjanović and Laurin (2007: Figure 5).

**FIGURE 3:**
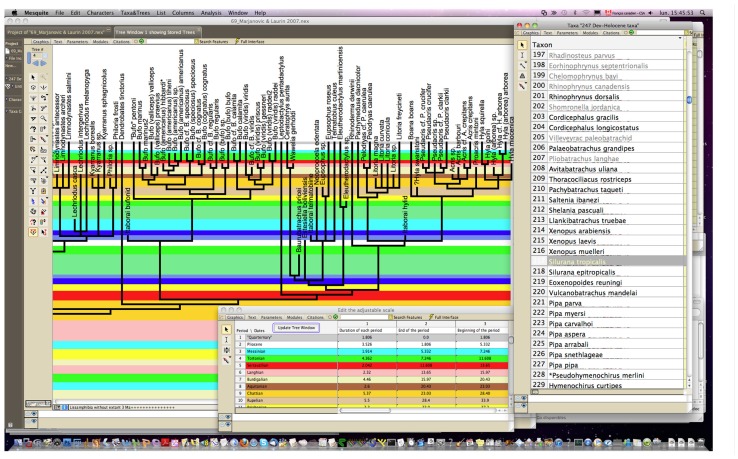
**Interface of Stratigraphic Tools (Josse et al., 2006) for Mesquite** (Maddison and Maddison, 2011). Screen capture showing a small part of a paleontological timetree. The geological timescale is user-specified through a dialog window (shown at the bottom) in Stratigraphic Tools; users need only enter name and duration of each period. The timescale can then be superposed onto the tree (main window); note that under the tree, the height of each period is proportional to its duration. Only the right window showing the taxon list is from the stock version of Mesquite.

Obtaining dating constraints is now the most severe bottleneck limiting our ability to accurately date the TOL. Various initiatives have been undertaken to solve this problem. A few paleontologists have compiled databases that can be used to set divergence time constraints (minimal as well as maximal). Thus, Benton (1993) published a large but family-level compendium, “The Fossil Record,’’ which has been widely used to provide minimal divergence dates. More recently, he has published a few papers (Benton and Donoghue, 2007; Donoghue and Benton, 2007; Benton et al., 2009) that provide several calibration constraints in a wide range of metazoans. Obviously, there is a real need for such papers, as shown by the high number of ISI citations of these papers [225 for Benton and Donoghue (2007)] according to the ISI databanks, searched on 13-7-2011). Reisz and Müller (2004) and Müller (2005) published more sharply focused papers documenting in detail the age of some divergences in amniotes and proposed some maximal ages for these divergences, although some molecular systematists disagreed with some of the maximal estimates (Hedges et al., 2006). Similarly, Marjanović and Laurin (2007) provided a time-calibrated supertree (a tree assembled from various other previously published trees) of lissamphibians showing the phylogenetic position (and geological age) of 223 extinct lissamphibian taxa based on their geological record, and of about 100 extant lissamphibian taxa, to show the minimal divergence dates between many extant lissamphibian taxa implied by their fossil record (**Figure 2**). This compilation was facilitated by new Mesquite modules that allow users to superimpose a geological timescale onto a phylogenetic tree (**Figure 3**) and provide several tools adapted to manipulating branches that represent extinct taxa (Josse et al., 2006). For instance, one such tool allows the user to move a taxon in the tree without changing the geological age (represented by the branch tip). Comparable software was recently developed for the R package (Ezard and Purvis, 2009). More recently, a project has recently been started to help centralizing dating constraint data and encourage gathering of such data (Ksepka et al., 2011).

### STRATIGRAPHY-BASED METHODS

Given that maximal age constraints are much more difficult to assess than minimal age constraints, the rest of this review will focus on how to obtain realistic maximal age constraints. Strauss and Sadler (1989) and Marshall, 1990, 1994, 1997, Marshall’s (2008) have published several papers that developed new methods to compute confidence intervals (CIs) on the true stratigraphic range of taxa. These methods can in principle be used to assess the maximal and most plausible divergence date between extant taxa, but such applications are hampered by various limitations of the methods. For instance, the method proposed by Strauss and Sadler (1989) and Marshall (1990) requires a random fossil discovery potential through time, which largely limits its field of applicability to species and small clades. However, when its assumptions are met, the method is very useful because CIs can be calculated easily, and their width depends simply on the observed stratigraphic range and on the number of horizons in which the taxon has a fossil record. Thus, if a taxon is represented by only two finds, the 95% CI is nearly 29 times larger than the observed range, but if 300 horizons contain the taxon, its 95% CI extends its observed range by only 1% (Marshall, 1990: Table 1).

A subsequent method (Marshall, 1994) allows for temporal variations in that discovery potential (as under an aggregative distribution of temporal distribution of fossil finds, for instance), but not increasing or decreasing trends. That method is based on a binomial distribution of temporal gaps, which are ordered by increasing size. Then, CIs can be computed if the gaps are numerous enough, and a particularity of this method is that the CIs themselves have CIs. For instance, if there are 17 gaps (18 fossil finds), the 95% CI of the 80% CI on the true stratigraphic range of the taxon may be as short as the 10th shortest gap, but it may also be as long as the 17th shortest (the longest) gap. Thus, this method requires a far denser fossil record than the method that assumes a random temporal distribution of fossil finds, and a 95% CI on the 95% CI of the stratigraphic range can be computed only if there are at least 72 gaps (73 fossil finds). This method is also inapplicable to large, diversifying clades.

**FIGURE 4:**
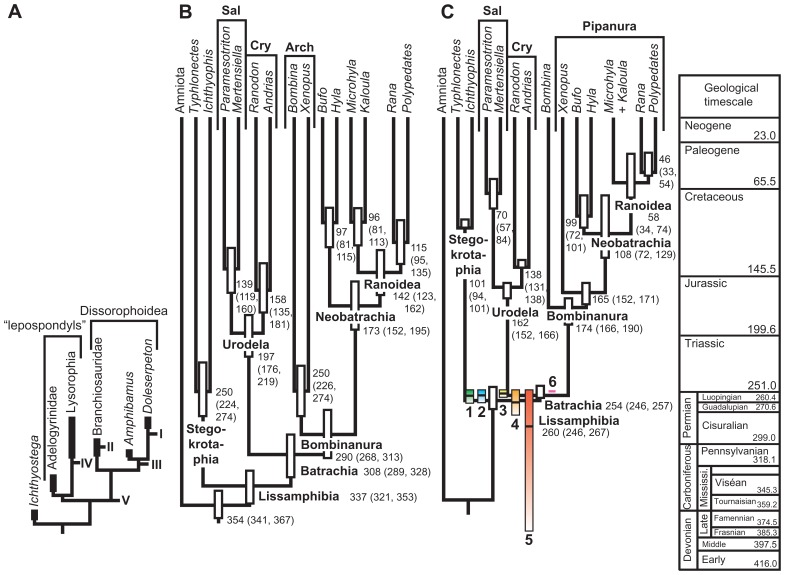
** Age of origin of Lissamphibia.** **(A)** Various branches that may have given rise to the lissamphibian stem in the Paleozoic at points I through V. Under hypotheses of polyphyly, extant amphibians derive from both lepospondyls (at least for gymnophionans) and dissorophoid temnospondyls (anurans and, typically, urodeles). **(B)** Molecular ages of various lissamphibian clades proposed by Zhang et al. (2005). Bars represent 95% credibility intervals. **(C)** Colored bars: confidence intervals on the age of appearance of lissamphibians, given the stratigraphic distribution of fossiliferous localities that have yielded lissamphibians, and assuming an exponential diversification model and various assumed extinction levels of lissamphibians resulting from major biological crises (colored bars 1–4). A model that assumes no impact of major crises on lissamphibians yields a much longer confidence interval (bar 5), whereas one assuming no diversification yields a ridiculously short interval (bar 6). Each bar represents a 75% CI, and the black line in the bar represents the 50% CI. The background tree comes from Marjanović and Laurin (2007) and shows ranges of ages based on different assumptions about minimal branch lengths. Reproduced from Marjanović and Laurin (2008).

A third method by Marshall (1997) is free of any assumptions about temporal distribution and can, in principle, be applied to any taxon. The method requires building a fossil recovery potential curve, which must be obtained independently from the stratigraphic distribution of fossils; this is required because otherwise the curve would fall to 0 on either end of the known distribution and CIs could not be computed. However, the curve must adequately predict the temporal occurrence of fossil finds; this can be tested by looking at the proportion of temporal variance explained by the curve. The particular criterion to be used to build the curve is obviously taxon-dependent. Marshall (1997) suggested that for some aquatic organisms, water depth could be used, as many aquatic benthic organisms have a reasonably narrow range of preferred depths, but such criteria are presumably useful for individual lineages of fairly small clades because large clades typically display a variety of environmental preferences (Barnes, 1987). For larger clades, Marshall (1997) suggested using the exposed surface area of appropriate sedimentary rocks. However, this criterion does not always work for large clades, as shown by the application of the method to the largest clade on which it has been applied so far, namely Lissamphibia. In that case, using exposed areas of continental sedimentary rocks predicted less than 6% of temporal distribution of fossil finds. This large extant clade has undergone strong diversification since its origin; thus, the discovery potential curve needs to reflect this to adequately predict the temporal distribution of fossil finds. Indeed, standing biodiversity of Lissamphibia evolved from two (at the origin, by definition) to more than 6000 species (extant biodiversity). Marjanović and Laurin (2008) showed that the temporal distribution of lissamphibian-yielding fossiliferous localities is explained mostly (*R*^2^ > 86%) by the paleobiodiversity of lissamphibians as inferred using a simple exponential diversification model. Incorporating the putative effect of mass extinction events (based on statistics from other taxa) such as the Permian/Triassic, Triassic/Jurassic, and the Cretaceous/Paleogene events (Jablonski, 1994) increased the explained variance a little further, to nearly 90%. But the generality of this result has to be verified by other empirical studies. For other taxa, other models (logistic, linear, or density-dependent) might possibly be more suitable. The main limitation of this approach is that the fossil recovery potential function is poorly constrained, and it can have an important impact on the resulting CIs (**Figure 4**). Nevertheless, application of this method to the lissamphibian fossil record suggests that lissamphibians appeared in the Permian (**Figure 4**), even though this depends partly on poorly constrained extinction levels of lissamphibians across major extinction events, such as the Permian/Triassic (Ward et al., 2005) or the Cretaceous/Paleogene (Wilf and Johnson, 2004) crises.

**FIGURE 5:**
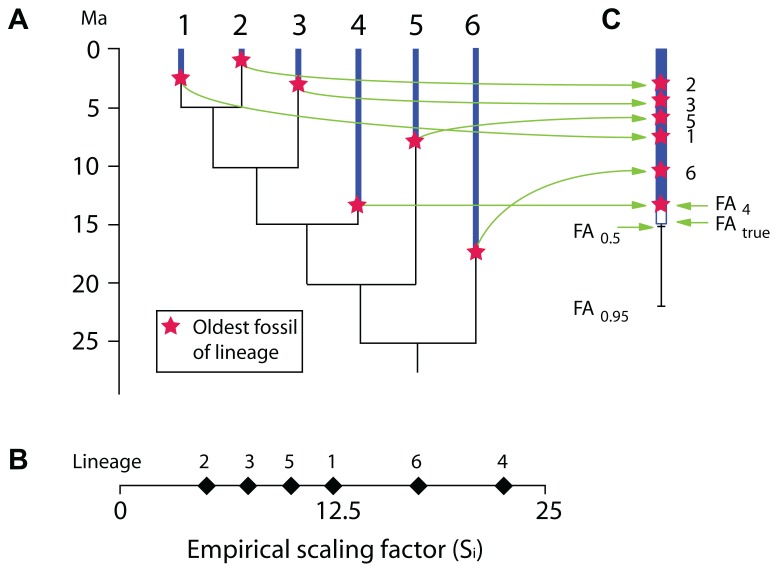
** Hypothetical example illustrating the basic principles of Marshall’s (2008) method for calculating confidence intervals on the appearance of taxa based on their fossil record and an uncalibrated ultrametric molecular tree.** **(A)** Ultrametric molecular tree with stratigraphic range of known fossil record of each terminal branch. **(B)** Empirical scaling factor (S_i_) for each of the six terminal lineages. In this hypothetical example, the true (unknown) root age is 25 Ma. **(C)** Same, shown vertically, along with the 50 and 95% confidence intervals on the first appearance (FA) of the lineage with the highest S_i_ (lineage 4) if we assume that a single fossiliferous horizon is known for each of the six terminal lineages.

Most recently, Marshall’s (2008) proposed a method to estimate the maximal age of a clade. That method is designed especially to allow fixing a maximal age of a clade represented in a molecular tree in order to provide a calibration constraint (a maximal age). It requires an uncalibrated molecular timetree (an ultrametric tree obtained without calibration constraints, typically using a relaxed-clock algorithm) and knowledge of the oldest fossil records of all lineages present in the molecular tree (**Figure 5A**). From these two sources of data, an empirical scaling factor (S_i_) is computed for each branch (**Figure 5B**). S_i_ is simply the age of the oldest fossil from each branch (A_i_) divided by the relative length L_i_ of each branch (L_i_ could vary between 0 and 1 if the height of the uncalibrated tree were initially set to 1). This ratio is then used to select the branch with the most complete fossil record, which is simply the branch with the greatest S_i_. Thus, in **Figure 5**, lineage 4 has the greatest S_i_ because its oldest fossil is from 13.5 Ma ago and L_i_ is 0.6, which yields a S_i_ of 13.5/0.6 = 22.5, which implies that the root is at least 22.5 Ma old. However, this is obviously an underestimate (this root node could be an unbiased estimate if lineage 4 had truly appeared at 13.5 Ma ago, in other words, if its stratigraphic coverage were perfect), and in the hypothetical example, the true root age would be 25 Ma (**Figure 5A)**. Therefore, Marshall’s (2008) proposes various formulae that can be applied to derive a CI on the age of calibration lineage and hence of the root, whether or not information is available about the number of fossiliferous horizons (when unknown, a single horizon is presumed known for each lineage, which yields broader CIs than if more horizons are known, as is nearly always the case). If we assume that a single horizon is known for each lineage, we have:

$$\begin{array}{cc} S_{c}=S_{cal}/{\left(1-C\right)}^{1/n}, & \left(1\right) \end{array}$$

where S_c_ is the scaling factor for a CI *C*, S_cal_ is the scaling factor of the calibrating lineage (with the greatest S_i_), *C* is the CI for which we want to compute S, *n* is the number of lineages.

Thus, in our hypothetical example, if we wanted to compute a 95% CI for the calibrating lineage age, Eq. 1 can be rewritten as: 

$$\begin{array}{cc} FA_{c}=FA_{cal}/{\left(1-C\right)}^{1/n}, & \left(2\right) \end{array}$$

where FA is the age of the first appearance of the lineage.

In this case, this gives us:

$$\begin{array}{c} FA_{c}=13.5/{\left(1-0.95\right)}^{1/6}=22.24\,\;Ma\,\;\left(Figure\,\;5C\right). \end{array}$$

If more than one fossil-bearing horizon is known for each lineage, the CIs are narrower, as expected:

$$\begin{array}{cc} FA_{c}=FA_{cal}/{\left(1-C\right)}^{1/nH}, & \left(3\right) \end{array}$$

where H represents the average number of fossiliferous horizons known for each lineage (this is the only difference from Eq. 2). Thus, if we know only two fossil-bearing horizons for each of the six lineages on average, in the same hypothetical example, the 95% CI on the appearance date of the calibrating lineage would be:

$$\begin{array}{cc} FA_{c}=13.5/{\left(1-0.95\right)}^{1/6*2}=17.33\,\;Ma. & \left(4\right) \end{array}$$

This method is operationally simple and requires few data. It is even possible to verify the reliability of the stratigraphic data by using a Kolmogorov–Smirnov test to remove fossils that are obviously too old to fit in a given lineage. For instance, a fossil dating from 15 Ma ago and reported as pertaining to lineage 1 (**Figure 5**) would most likely be misattributed, and a Kolmogorov–Smirnov test could show that.

Unfortunately, Marshall’s (2008) method requires a uniform (random) discovery potential for the taxa represented by the terminal branches through time, which is an unrealistic assumption for any diversifying clade because the preservation potential of a clade is expected to increase with its diversity, geographic range, and number of individuals, all of which are lowest at its origin and extinction. Even for clades that have not diversified much, preservation may be non-random because environmental fluctuations, such as sea-level changes, and subsequent erosion of sediments leave large and not necessarily random gaps in the fossil record. This assumption of random fossilization potential through time limits its field of application to cases in which the terminal branches represent individual lineages or small clades (each of which should have only a few extant species). The analyses of Marjanović and Laurin (2008) showed to that the fossilization potential of large clades such as Lissamphibia could be easily modeled based on their inferred paleobiodiversity. However, the generality of this problem has not been verified, and Marshall’s (2008, p. 738–739) discussed this caveat in detail and proposes various solutions.

Another limitation of the approach is that it rests on the assumption of clock-like changes in the molecular data, an assumption that is known to be violated to a variable extent in most genuine datasets. The advantage of the relaxed-clock methods is precisely that they can use multiple calibration constraints to model variations in molecular evolutionary rates (Sanderson, 2002), but using Marshall’s (2008) method, these variations cannot be taken into consideration given that the tree is uncalibrated, a limitation that Marshall’s (2008, p. 728) recognized. However, the effect of this factor can be assessed by comparing the relative branch lengths of an uncalibrated molecular tree with those of a tree obtained using multiple minimal and maximal age constraints; if both sets of length are similar, the lengths of the uncalibrated tree should be reliable.

### BIRTH-AND-DEATH PROCESSES

**FIGURE 6:**
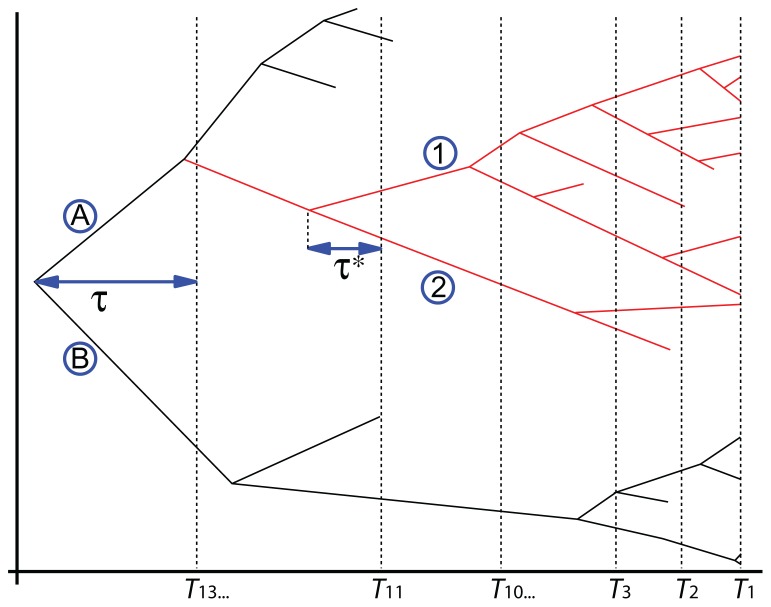
** A primate tree used by Wilkinson and Tavaré (2009) to explain their method and to obtain probability distributions on the times of origin of anthropoids (clade in red) and crown-group primates.** The oldest anthropoid dates from T11 and the oldest crown primate from T13 (Table 1). Thus, the unknown gaps in the earliest fossil record of primates and anthropoids, here denoted τ and τ *, respectively, are the time intervals that need to be estimated to know the true times of origin of these two clades. The four clades denoted by alphanumeric symbols are: A, Haplorhini; B, Strepsirhini; 1, Platyrrhini; 2, Catarrhini.

Recently, theoretical biologists have produced and used mathematical models to study the diversification rate of taxa (e.g., Patzkowsky, 1995; Przeworski and Wall, 1998; Reed and Hughes, 2007; Crisp and Cook, 2009), and such methods can also yield information about divergence times. For instance, Foote et al. (1999a) showed that the early (Cretaceous) age of origin of many placental groups implied by several molecular studies was unlikely. This finding is based on the minimal number of lineages that must have existed and estimates of preservation potential obtained from other studies. Similarly, Wilkinson and Tavaré (2009) used conditioned birth-and-death processes to estimate the probability that primates and anthropoids existed in the Cretaceous. Their method requires information only about the known biodiversity of primates and anthropoids in various time intervals (including their FA in the fossil record). It requires no morphological and no molecular data, although such data must have been used (by others) to assess the affinities of extinct and extant taxa to produce the paleobiodiversity data that are used by the method (**Figure 6**). It rests on the hypothesis that rates of speciation (here equated with cladogenesis) and extinction are constant through time and among lineages. Fossilization is also modeled as a Poisson process, which describes which branches of simulated trees are represented in the fossil record. The fossil sampling rate is allowed to vary through time (Wilkinson and Tavaré use 14 time bins in their example; see Table 1), which accounts for unevenness in the richness of the fossil record. The process can then be conditioned on having two clades (in the example, crown-primates and crown-anthropoids) originate no later than a given time (chosen to match the oldest known fossil of each clade) and the simulated trees are retained if they reasonably closely match the observed number of fossils in the various time bins and the observed extant biodiversity (**Figure 6**). These simulated trees can then be used to establish the posterior distribution of true origination times of the clades of interest. Wilkinson and Tavaré (2009) thus showed that the fossil record of primates is compatible with an appearance of the clade in the Mesozoic because the 95% credibility interval for the origin of crown-primates (euprimates) encompasses the range from 54.8 to 98.9 Ma ago. This analysis cannot refute the molecular dates of origin of many mammalian clades (Bininda-Emonds et al., 2007; Meredith et al., 2011), often considered too old by paleontologists (e.g., Foote et al., 1999a, 1999b), given what we know about the fossil record of primates. This partly reflects the fact that this method yields (at least with this dataset) very broad credibility intervals. However, for anthropoids, the 95% CI extends only to 53.2 Ma ago. More recently, Wilkinson et al. (2011) developed an integrated approach that uses a similar method to establish priors on divergence times and uses that information in molecular dating to get a posterior divergence time distribution. 

**Table 1:** Number of primate and anthropoid species that are extant or known from the fossil record used in the example of Wilkinson and Tavaré (2009). Geological time is divided into 14 intervals (k).

Epoch	k	Time at base of interval k(Ma)	Primate species counts, D	Anthropoid species counts,A
Extant	0		376	281
Late Pleistocene	1	0.15	22	22
Middle Pleistocene	2	0.9	28	28
Early Pleistocene	3	1.8	30	30
Late Pliocene	4	3.6	43	40
Early Pliocene	5	5.3	12	11
Late Pliocene	6	11.2	38	34
Middle Miocene	7	16.4	46	43
Early Miocene	8	23.8	34	28
Late Oligocene	9	28.5	3	2
Early Oligocene	10	33.7	22	6
Middle Eocene	12	49.0	119	0
Early Eocene	13	54.8	65	0
Paleocene and earlier	14		0	0

### COMBINED MOLECULAR AND PALEONTOLOGICAL DATING

Another, complementary approach consists in integrating extinct taxa into combined (molecular and morphological) trees that are dated using standard techniques developed for molecular sequences. An early step into this direction consisted in a study of Juglandaceae (walnut family) that incorporated five extinct and 25 extant taxa into a combined molecular and morphological analysis to place extinct taxa into a phylogeny and infer minimal ages of various clades (Manos et al., 2007). Lee et al. (2009) went a bit further by integrating uncertainty about the phylogenetic position of the extinct squamate *Pygopus hortulanus* (about 20 Ma) in a molecular dating study of Pygopodidae through a Bayesian analysis. For this, they added 19 mandibular characters to their molecular matrix (the fossil is represented only by a mandible). They showed that despite the fact that the position of *P. hortulanus *recovered by their combined analysis is the previously suggested one, other positions were recovered in some trees. This resulted in a fourfold increase in the width of the credibility intervals of the molecular ages of the various clades, demonstrating the importance of incorporating topological uncertainty about the calibrating lineages into such analyses. Magallón (2010) was the first study that tried to assess branch lengths of extinct taxa to determine how this would influence molecular dates. She inserted 13 extinct terminal taxa into a molecular and morphological matrix that also included 70 extant embryophyte taxa. The purpose of that study was to determine if the long branch subtending crown-angiosperms explained that the previous molecular dates for that node were much greater than what is suggested by the fossil record. She performed a total evidence parsimony analysis (the molecular characters were scored as unknown for the extinct taxa) to obtain an initial topology and set of branch lengths. She then adjusted the branch lengths (initially reflecting inferred amount of change in the molecular sequences) of the extinct taxa to take into consideration their known stratigraphic range and their possible divergence time from the stem of extant taxa, and simulated 100 molecular datasets for these extinct taxa using evolutionary models selected on the basis of the molecular sequences of the extant taxa on a tree (a maximum a posteriori tree). She then performed several molecular dating analyses using a variety of methods and concluded that the long branch subtending the basal node of crown-angiosperms does not explain the old molecular age inferred fort his node. That study made greater use of the fossil record than many previous molecular dating analyses, but it rested on several assumptions (Magallón, 2010, p. 394), including that the molecular evolutionary rates of extinct taxa was the average of the rate inferred for extant taxa and that the divergence times between these extinct taxa and extant ones had been correctly determined. These assumptions are admittedly tentative, especially the first one.

Pyron (2011) dealt differently with extinct taxa. Instead of simulating molecular data for these, which was probably the most speculative step in the analyses of Magallón (2010), lengths of all branches are assessed simultaneously. For extinct taxa, these are usually determined by using morphological data only. However, this approach can also be used for molecular sequences that come from serially sampled viruses or ancient DNA (Ho and Phillips, 2009). This allows incorporating phylogenetic uncertainty affecting extinct taxa into the analysis, contrary to the traditional approach in which the position of the oldest fossil of a clade must be considered as known without error, at least with respect to the other terminal taxa represented by molecular sequences in a given analysis. This approach is now conveniently implemented in the latest version (3.2) of MrBayes (Ronquist et al., 2012), which now performs molecular dating and allows constraints to be applied to internal nodes (the classical approach) as well as to terminal taxa, which allows incorporation of extinct taxa directly into the timetree.

This approach is extremely useful, although as implemented by Pyron (2011), it requires application of model-based methods developed for molecular data to morphological data, for which the justifications for such applications are more tenuous than for molecular data. For instance, there is no equivalent in morphology to the natural partitions of molecular datasets into first, second, and third codon positions, between nuclear and mitochondrial genes, and between introns and exons. Morphological characters are much more complex than molecular characters (typically positions of single nucleotides or amino acids), which reduces the probability that they evolved according to simple models. Furthermore, branch lengths for extinct taxa need to be estimated using the sometimes very limited anatomical information provided by the fossil record. The main practical problem with this approach is that the morphological part of the mixed matrix needs to include a random sample of characters evolving on all branches. Such a sampling has rarely been attempted to my knowledge; a few exceptions include and Müller (2006), Brusatte et al. (2008), and Organ et al. (2011b). On the contrary, morphological data matrices are usually compiled to resolve a given phylogenetic problem using parsimony, with a taxon and character sampling reflecting this preoccupation. Thus, autapomorphies of terminal taxa and other parsimony-uninformative characters are generally excluded from such matrices, and this may bias branch length estimates. Also, given that building large morphological data matrices is cumbersome (there is no morphological equivalent of GenBank for molecular sequences, unfortunately), this method may be applicable mostly to small and mid-sized trees, although this will need to be assessed in future studies. Nevertheless, this method has the advantage of using the most detailed available data (molecular sequences, morphological data, the resulting topologies, geological age of fossils, and branch lengths inferred on the basis of all these data) of all methods surveyed here.

**FIGURE 7:**
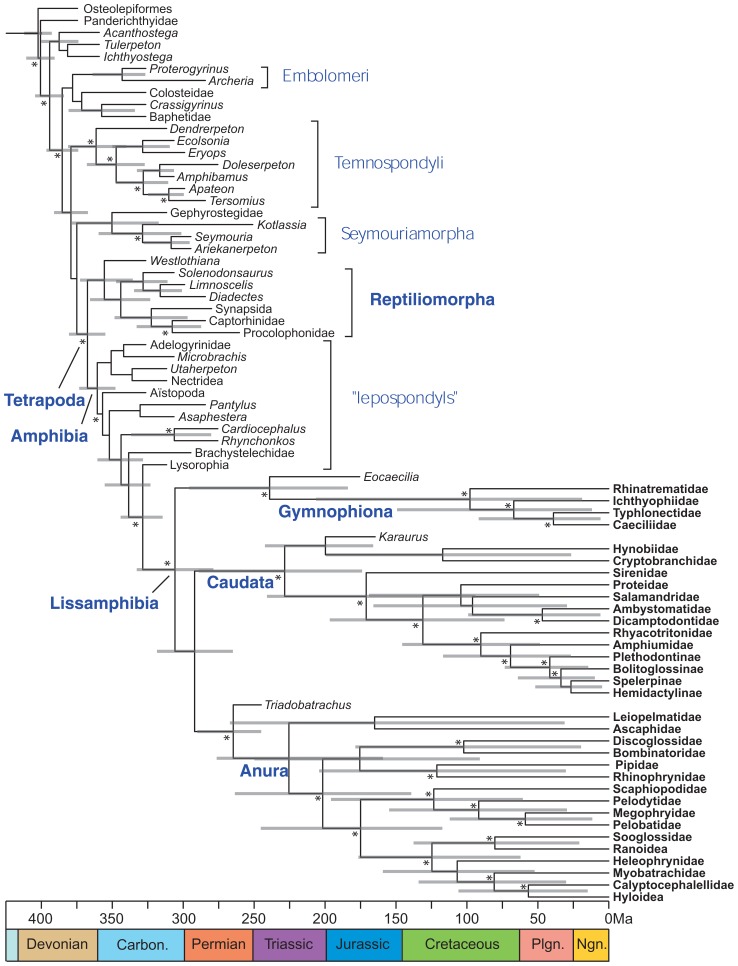
**Combined molecular and morphological timetree of limbed vertebrates.** Modified from Pyron (2011). Gray bars represent 95% highest posterior density. Nodes without bars are represented by less than 50% posterior probability (Pp). Nodes supported by Pp > 95% are marked by an asterisk (*). Extant taxa are in bold type. Carbon, Carboniferous; Ngn, Neogene; Plgn, Paleogene

Application of this method to lissamphibians (Pyron, 2011) has already clarified their origins, to the extent that lissamphibians appear to be monophyletic, to be nested within lepospondyls, and to have originated at the earliest in the Late Carboniferous (**Figure 7**), whereas previous molecular dating had suggested much earlier origins (in the Late Devonian or Early Carboniferous) that was more coherent with lissamphibian polyphyly, with respect to their prospective Paleozoic relatives (San Mauro et al., 2005; Lee and Anderson, 2006; Roelants et al., 2007; Marjanović and Laurin, 2007).

## PERSPECTIVES

Time is ripe for investigating how to better date the TOL using paleontological data because paleontological phylogenetics has undergone a revolution through the advent of cladistics, and this has triggered a tremendous growth in the number and reliabilities of paleontological trees. This revolution is more recent than it might first appear. Although Hennig (1966) introduced cladistics over 50 years ago, his German book was read by few and understood by fewer still (if only, perhaps, due to the facts that Hennig published in East Germany and in a highly technical, philosophical style). The English translation (Hennig, 1966) made the text accessible to far more scientists, and the method slowly gained popularity in the systematic community over the next three decades. However, adoption of cladistics proceeded at a different pace in each field, with human paleontology, for instance, lagging far behind the study of Paleozoic vertebrates, although thorough studies have now been performed in both fields (e.g., Strait and Grine, 2004; Müller 2006). Nevertheless, all fields of paleontology have produced well-corroborated phylogenies supported by data matrices over the last decade, and the increasing power of microcomputers and sophistication of phylogenetic analysis software (Swofford, 2003; Goloboff et al., 2008) now allow paleontologists to incorporate several dozen (e.g., Marjanović and Laurin, 2009), if not hundreds of taxa simultaneously (e.g., Ruta and Coates, 2007). All this has resulted in a fairly large but scattered amount of underused paleontological data in the form of phylogenies that could be time-calibrated and assembled into supertrees to yield new insights to date the TOL, using birth-and-death models, for instance. This recent progress also allows implementing more rigorous standards for defining calibration constraints, as requested by several authors (e.g., Parham et al., 2012).

Three limiting factors currently hamper progress in dating the TOL: the lack of extensive paleontological timetrees (that are essential to document minimal divergence times) or compilations of paleontological dating constraints, more sophisticated analytical methods based on birth-and-death processes that could better exploit paleontological timetrees to extract divergence time constraints, and the paucity of datasets suitable for mixed paleontological and molecular dating. Recent developments should help solve the first problem because Stratigraphic Tools (Josse et al., 2006) and paleoPhylo (Ezard and Purvis, 2009) facilitate paleontological timetree construction, whereas the initiative supported by Palaeontologia Electronica (Ksepka et al., 2011) should promote compilation of a large database of divergence time constraints. However, there is still no method to fully exploit paleontological timetrees to get statistically validated temporal distributions of the probability of appearance times of clades, although the method developed by Wilkinson and Tavaré (2009) can use part of these data. Using birth-and-death processes, a method using paleontological timetrees to extract data on the time of origin of taxa could in principle be implemented, so it is probably only a matter of time till paleontological data regain their central role in dating the TOL and lead to substantial improvements in the accuracy of what can be achieved by molecular dating. And last but not least, it will be interesting to see how widespread combined paleontological and molecular dating will become in the coming years. Through a combination of all these advances, dating the TOL is likely to become much easier.

## Conflict of Interest Statement

The author declares that the research was conducted in the absence of any commercial or financial relationships that could be construed as a potential conflict of interest.
